# Survival and Environmental Stress Resistance of *Cronobacter sakazakii* Exposed to Vacuum or Air Packaging and Stored at Different Temperatures

**DOI:** 10.3389/fmicb.2019.00303

**Published:** 2019-02-20

**Authors:** Yichen Bai, Haibo Yu, Du Guo, Shengyi Fei, Chao Shi

**Affiliations:** College of Food Science and Engineering, Northwest A&F University, Yangling, China

**Keywords:** *Cronobacter sakazakii*, air packaging, vacuum packaging, survival, environmental stress

## Abstract

The aim of this study was to evaluate the survival of *Cronobacter sakazakii* exposed to vacuum or air packaging, then stored at 4, 10, or 25°C, and the environmental stress resistance of vacuum-packaged or air-packaged bacterial cells were determined by subjecting the cells to reconstituted infant formula at 50°C, in acid (simulated gastric fluid, pH = 3.5), and in bile salt [bile salt solution, 5% (wt/vol)]. A cocktail culture of *C. sakazakii* desiccated on the bottom of sterile petri plates was air-packaged or vacuum-packaged and then stored at 4, 10, or 25°C for 10 days. The viable cell populations during storage were examined, and the vacuum-packaged and air-packaged cells (stored at 10°C for 4 days) were subsequently exposed to heat, acid, or bile salt. The results show that the populations of vacuum-packaged and air-packaged *C. sakazakii* were reduced by 1.6 and 0.9 log colony-forming units (CFU)/ml at 4°C and by 1.6 and 1.3 log CFU/ml at 25°C, respectively, in 10 days. At 10°C, significant reductions of 3.1 and 2.4 log CFU/ml were observed for vacuum-packaged and air-packaged cells, respectively. Vacuum packaging followed by storage at 10°C for 4 days caused significant decreases in the resistance of *C. sakazakii* to heat, acid, and bile salt conditions compared with air packaging. These results suggest that the application of vacuum packaging for powdered infant formula could be useful to minimize the risk of *C. sakazakii*.

## Introduction

*Cronobacter sakazakii* (formerly known as *Enterobacter sakazakii*) is a Gram-negative, non-spore-forming bacillus that exists in the environment as well as in a wide variety of foods. It is regarded as a newly developing foodborne pathogen (Wan-Ling et al., 2010). *C. sakazakii* has been implicated in severe forms of neonatal infection, such as bacteremia, meningitis, and necrotizing enterocolitis, particularly in infants and premature babies; the mortality rates associated with these bacteria range from 50 to 80% (Li et al., 2013). Powdered infant formula (PIF), which is a main source of nutrition for neonates and infants, has been recognized as the major vehicle of transmission of *C. sakazakii*, and the consumption of contaminated PIF is associated with the majority of *C. sakazakii* outbreaks (Shukla et al., 2016). Due to the role of trehalose accumulation within its cells, *C. sakazakii* is remarkably resistant to desiccation (Breeuwer et al., 2010). Some capsulated strains of *C. sakazakii* in dehydrated PIF (with a low water activity of 0.2) were still recoverable after 2.5 years (Barron and Forsythe, 2007). Fei et al. (2017) determined the prevalence of *C. sakazakii* isolates from PIF collected from Chinese retail markets, and the contamination rate of *C. sakazakii* in that study was 2.8%. Heperkan et al. (2017) reported in a different study that the prevalence of *C. sakazakii* was 8.0% in milk powders and that the number of *C. sakazakii* was 7–15 MPN/g.

Foodborne pathogens face several hurdles after they enter the host. The strong acid condition in the stomach is the first line of host defense against these pathogens. Additionally, the presence of bile salts, antimicrobial peptides, and other hostile conditions also act as defenses against serious infections in humans (Anf et al., 2017). *C. sakazakii* exhibits unusual resistance to acid stress growth conditions, and they can grow at minimum pH values of ∼4.5, although this value varies depending on the strain and type of acid (Alvarezordóñez et al., 2014). Bile is an important antimicrobial component of the human digestive system, but growth has been described for some *C. sakazakii* isolates at bile salt concentrations as high as 5% (Fakruddin et al., 2014). Compared with other members of the Enterobacteriaceae family, *C. sakazakii* is reported to be significantly more thermotolerant (Amalaradjou and Venkitanarayanan, 2011). This feature represents a competitive advantage, facilitating its survival during improper PIF reconstitution (Shi et al., 2017).

Environmental stresses are known to induce adaptive responses within the bacterial cell. Bacterial pathogens have the ability to enhance their resistance to lethal stresses after their exposure to a sublethal one via genetic regulation or physiological adaptation (Yang et al., 2015). The phenomenon of one type of stress-response imparting auxiliary protection to cells subsequently stressed at higher levels is widely documented and may be referred to as “cross-protection” (Wesche et al., 2009). Cross-protection has been a growing concern in the microbiological food safety area.

To reduce the levels of *C. sakazakii* contamination in the finished products reconstituted from PIF, which are introduced during food processing, many thermal and non-thermal technologies for *C. sakazakii* inactivation have been proposed (Ha and Kang, 2014; Pina-Pérez et al., 2015; Shi et al., 2016). Additionally, the Food and Agriculture Organization of the United Nations/World Health Organization (2007) recommended that PIF reconstitution should be performed at 70°C to reduce the risk of *C. sakazakii* survival during PIF preparation, and some studies have investigated the efficacy of antimicrobials for reducing the tolerance of *C. sakazakii* to environmental stresses to improve the safety of PIF (Amalaradjou and Venkitanarayanan, 2011). However, studies focusing on factors affecting the survival and environmental stress resistance of *C. sakazakii* during the packaging and storage processes are lacking.

The aim of this work was to evaluate the survival of *C. sakazakii* exposed to vacuum or air packaging and stored at 4, 10, or 25°C. Additionally, the effects of vacuum packaging and air packaging on the thermotolerance and survival under simulated gastrointestinal conditions and bile salt conditions of *C. sakazakii* strains were also assessed. Desiccated *C. sakazakii* was used in this study to simulate the conditions of intrinsic PIF contamination.

## Materials and Methods

### Bacterial Culture

Three strains of *C. sakazakii* (ATCC 29544, 14-15, and 18-8) were used for this study. ATCC 29544 was procured from the American Type Culture Collection (ATCC, Manassas, VA, United States). Strains 14-15 and 18-8 were isolated from PIF and baby formula, respectively (Li et al., 2016). All *C. sakazakii* strains were stored at -80°C in Luria-Bertani (LB) broth with 30% (vol/vol) glycerin. To activate the frozen cultures, a loopful of each strain was streaked with a flamed loop onto Tryptic Soy Agar (TSA, Land Bridge, Beijing, China) and incubated at 37°C for 24 h, followed by incubation at 37°C in sterile Tryptic Soy Broth (TSB; Land Bridge) for 18 h.

### Preparation of Desiccated *C. sakazakii*

The method previously described by Al-Nabulsi et al. (2009) was used with slight modifications to prepare desiccated *C. sakazakii*. After centrifugation (8000 × *g*, 10 min, 4°C), each 18-h culture of *C. sakazakii* was washed twice with sterile phosphate buffered saline (PBS, pH = 7.4). Each culture was then adjusted to an OD_600__nm_ value of 1.0 with 0.2% (wt/vol) buffered peptone water (BPW). Equal volumes of three cultures were aseptically combined to produce a cocktail. Subsequently, 50 μl of this *C. sakazakii* cocktail was distributed evenly into the bottom of sterile petri plates. The plates were placed in a drying oven at 40°C for 2 h without their lids. The plates were then covered with lids, transferred to a desiccator, and stored at 25°C for 4 days to dry the cells.

### Preparation of Vacuum-Packaged and Air-Packaged *C. sakazakii*

For vacuum packaging, a vacuum sealer (Deli Group No. 14885, Zhejiang, China) was used. The plates coated with desiccated *C. sakazakii* were vacuum-packaged in thick plastic bags (Deli Group No. 14914) made of polyamide/polyethylene. For air packaging, the plates were placed in the same type of plastic bags and packed with air.

### Viability of Survivors During Storage

All packaged samples were stored at 4, 10, or 25°C, and viable cell populations during storage were examined on day 0, 1, 2, 3, 4, 5, 6, 7, 8, 9, and 10, respectively. To collect the desiccated *C. sakazakii*, 3 ml of 0.2% (wt/vol) BPW was added to the bottom of each sterile plate. Serial dilutions of the collected cells made using PBS, and 100 μl of the diluents were plated evenly in triplicate on TSA plates. Five 200-μl aliquots of the undiluted samples also were spread plated onto five TSA plates to achieve a detection limit of 3 CFU/ml. The number of colonies was counted after a 24-h incubation at 37°C.

### Effect of Vacuum and Air Packaging on the Environmental Stress Resistance of *C. sakazakii*

PIF was procured from a local supermarket (Yangling District, China). To detect any natural contamination, the method previously described by Yang et al. (2015) was employed with slight modifications, and background microflora (<25 CFU/g) was observed in the PIF, the colony morphology of background microbes was obviously bigger than that of *C. sakazakii*. The PIF (13.5 g) was then reconstituted with 90 ml of sterile deionized water as per the manufacturer’s instructions on the label. Solutions of desiccated cells without storage (control), desiccated cells subjected to vacuum packaging and stored for 4 days, or desiccated cells subjected to air packaging and stored for 4 days were collected, the bacterial concentration of each sample was adjusted, and the samples were added to the reconstituted infant formula. If necessary, several samples treated in the same conditions were pooled together to reach the fixed bacterial concentration for the experiment.

#### Determination of Heat Resistance of *C. sakazakii*

For heat resistance assay, the concentration of initial sample was about 3.0 log CFU/ml. All experimental samples were exposed to heat stress of 50°C in a water bath for 0, 10, 20, 30, or 60 min.

#### Determination of Acid Resistance of *C. sakazakii*

The acid resistance of *C. sakazakii* cells was determined by subjecting them to simulated gastric fluid (SGF, pH = 3.5). The SGF consisted of 8.3 g/L proteose-peptone, 2.05 g/L NaCl, 0.6 g/L KH_2_PO_4_, 0.11 g/L CaCl_2_, 0.37 g/L KCl, 0.05 g/L oxgall, 1 g/L lysozyme, and 13.3 mg/L of pepsin. All compounds were dissolved in deionized water and autoclaved together except for the oxgall, lysozyme, and pepsin, which were filter-sterilized (0.25 μm). The final pH was adjusted with a sterile 5.0 N HCl solution.

All experimental samples were separately mixed with SGF solution. To simulate the environment of the human body, the method previously described by Anf et al. (2017) was employed, the samples were placed in a shaker (130 rpm) at 37°C for different lengths of time (0, 10, 20, 40, or 60 min). For acid resistance assay, the bacterial concentration of initial sample was about 2.7 log CFU/ml.

#### Determination of Bile Salt Resistance of *C. sakazakii*

The bile salt resistance of *C. sakazakii* strains was determined according to the method previously described by Fakruddin et al. (2014) with slight modifications. The *C. sakazakii* solutions were transferred to 5% (wt/vol) bile salt solution and placed in a shaker (45 rpm) at 37°C for different lengths of time (0, 20, 60, 90, or 120 min). For bile salt resistance assay, the bacterial concentration of initial sample was about 2.7 log CFU/ml.

For each sampling time, viable cell populations were determined on three TSA plates by a spread plate method with serial dilutions made using PBS. All plates were incubated at 37°C for 24 h before the colonies were counted. The results were expressed as percentage of survived cells (%). Percentage of survived cells (%) = N_1_/N_0_, where N_1_ represented the CFU after heat, acid or bile salt resistance and N_0_ represented the initial contamination of inoculated reconstituted infant formula.

### Statistical Analysis

Mean values and standard deviations were obtained from three replicate experiments with duplicated plating (*n* = 3). The effects of different treatments during the storage period were appraised by a one-factor analysis of variance (ANOVA), and means were separated at the 95% confidence level (the difference was considered significant if *p* < 0.05) using SPSS software (Ver 12.0K, SPSS Inc., Chicago, IL, United States).

## Results

### Effects of Vacuum and Air Packaging on *C. sakazakii* Populations at 4, 10, and 25°C

**FIGURE 1:**
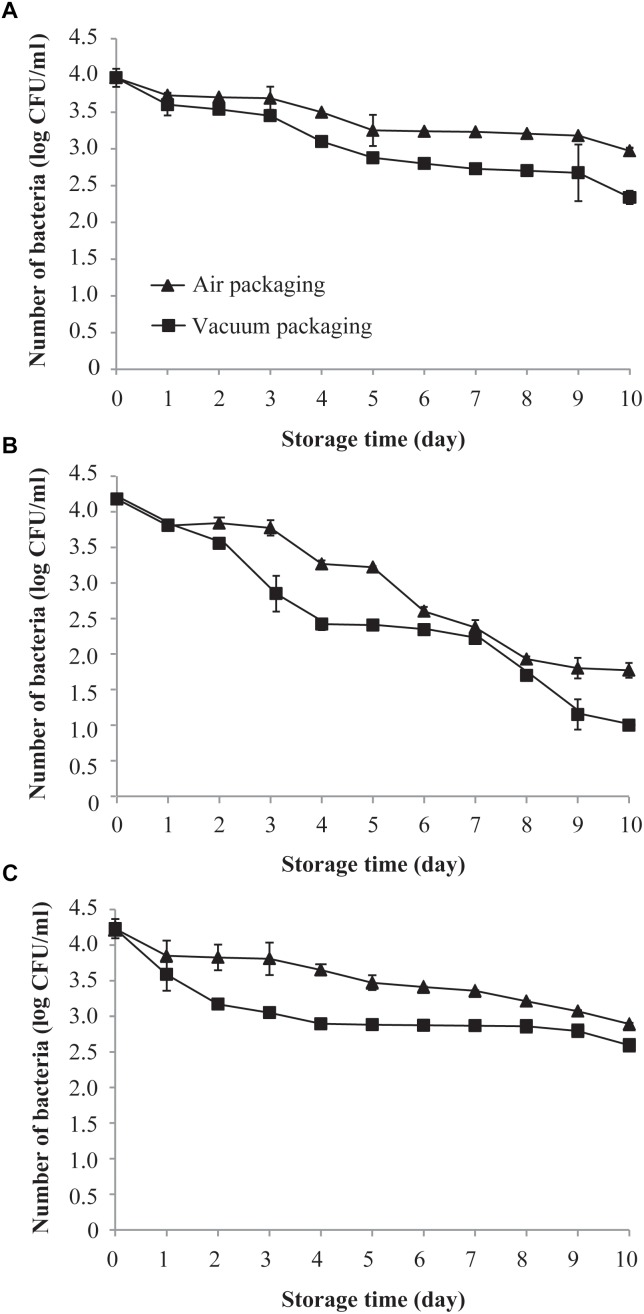
Effects of air packaging and vacuum packaging on the survival of *C. sakazakii*. **(A–C)** The survival of *C. sakazakii* subjected to air packaging or vacuum packaging was assessed at 4°C **(A)**, 10°C **(B)**, and 25°C **(C)**. Values are means ± standard deviation of three replicates.

Figure 1A shows the effects of air and vacuum packaging on the survival of *C. sakazakii* exposed to 4°C for a period of 10 days. The initial mean population of *C. sakazakii* on day 0 (before storage) at 4°C in both samples was approximately 4.0 log CFU/ml. The viable population of *C. sakazakii*, regardless of being subjected to vacuum packaging or air packaging, decreased over the length of the exposure period. However, a more drastic reduction in the viable population was found for the vacuum-packaged cells than for the air-packaged cells. After 10 days of exposure to 4°C, the air-packaged *C. sakazakii* showed a population reduction of 0.9 log CFU/ml compared with 1.6 log CFU/ml for the vacuum-packaged cells.

As shown in Figure 1B, the survival of both air- and vacuum-packaged *C. sakazakii* cells dropped rapidly during the initial 10 days of exposure to 10°C. After 5 days of storage, air- and vacuum-packaged *C. sakazakii* were each strongly reduced, by approximately 1.0 and 1.7 log CFU/ml, respectively. At the end of the storage period, the air- and vacuum-packaged *C. sakazakii* cells exhibited a population reduction of 2.4 and 3.1 log CFU/ml, respectively.

As shown in Figure 1C, the survival rates of both air- and vacuum-packaged *C. sakazakii* cells at 25°C were similar to those observed at 4°C. There was a sustained reduction in the population of air-packaged cells over the 10-day period, and the survival of vacuum-packaged *C. sakazakii* cells dropped rapidly during the initial 4 days of storage. After 10 days of storage, a smaller population reduction of 1.3 log CFU/ml was noted for the air-packaged *C. sakazakii* compared with that for the vacuum-packaged cells (1.6 log CFU/ml). Based on all these results, 10°C was chosen as the experiment temperature and 4 days was chosen as the storage time for all subsequent experiments.

### Effects of Vacuum and Air Packaging on the Heat Resistance of *C. sakazakii*

**FIGURE 2:**
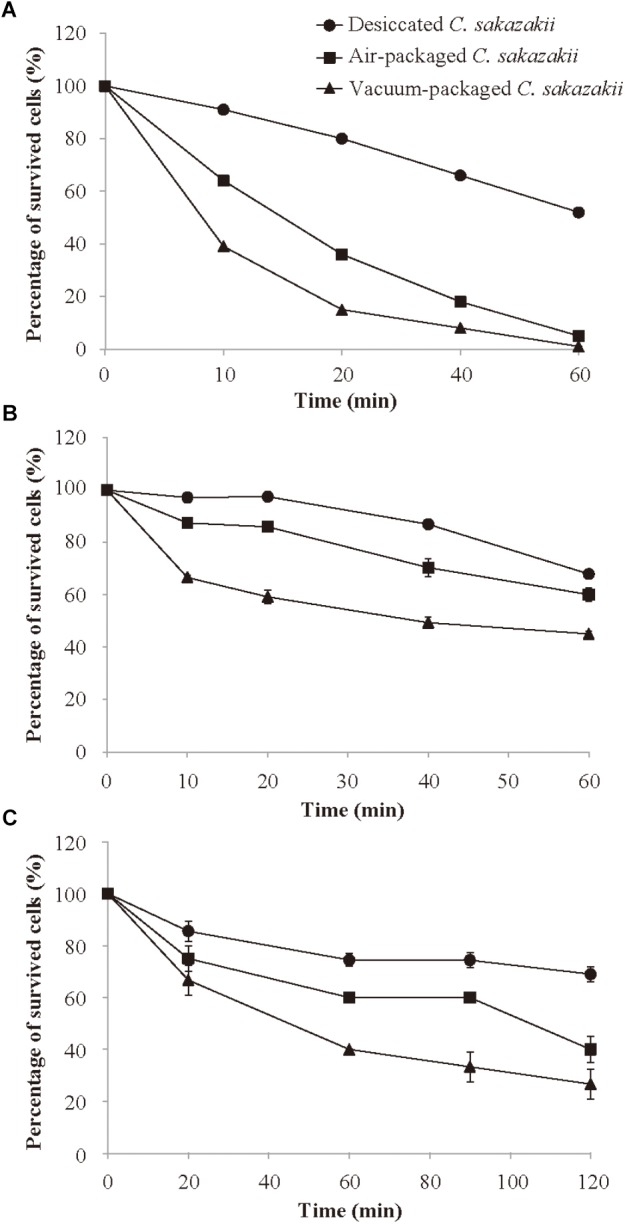
Survival of desiccated, air-packaged, or vacuum-packaged *C. sakazakii* in heat environment **(A)**, simulated gastric fluid **(B)**, and simulated bile salt **(C)**. Values are means ± standard deviation of three replicates.

After 4 days of storage at 10°C, *C. sakazakii* cells were subsequently subjected to 50°C to determine if air packaging and/or vacuum packaging influences the survivability of the pathogens at the regular temperature of PIF reconstitution. The number of *C. sakazakii* decreased steadily within 60 min at 50°C for each of the three different treatments (Figure 2A). Throughout the entire 60-min period at 50°C, the desiccated cells that had not been subjected to storage generally showed a significantly (*p* < 0.05) higher survival rate compared with the stored air- or vacuum-packaged cells. The vacuum-packaged cells were significantly more susceptible to heat (50°C) than were the air-packaged cells. After 10 min of the heat treatment, the vacuum-packaged and air-packaged *C. sakazakii* cells exhibited the survival rates of 39.0 and 63.8%, respectively. At the end of the 60-min incubation period, the vacuum-packaged and air-packaged *C. sakazakii* cells exhibited the percentage of survived cells (%) of 1.0 and 4.9%, respectively.

### Effects of Vacuum and Air Packaging on the Tolerance of *C. sakazakii* to Simulated Gastric Fluid (SGF)

SGF (pH = 3.5) was prepared for use in studying whether the different packaging methods affect the survival of *C. sakazakii* after host consumption. As shown in Figure 2B, the survival rate decreased steadily during the entire incubation period for each treatment type, but the survival rate of the vacuum-packaged *C. sakazakii* was significantly (*p* < 0.05) lower than that of the air-packaged cells at each measurement interval. At the end of the 60-min incubation period, the air-packaged cells showed a survival rate reduction of 39.9% compared with the larger survival rate reduction of 54.8% for the vacuum-packaged cells.

### Effects of Vacuum and Air Packaging on the Bile Salt Tolerance of *C. sakazakii*

To determine the effect of vacuum and air packaging on the bile salt tolerance of *C. sakazakii*, the stored, packaged cells were exposed to a bile salt challenge for 120 min. As shown in Figure 2C, the survival rate after 20 min of bile salt exposure of the desiccated *C. sakazakii* that had not been subjected to storage was significantly higher (*p* < 0.05) than those of the vacuum-packaged and air-packaged cells. The difference in the percentage of survived cells (%) between the vacuum-packaged and air-packaged cells became pronounced as the exposure period was lengthened. At the end of the 120-min bile salt exposure period, the air-packaged *C. sakazakii* exhibited a survival rate reduction of 59.0%, whereas a larger survival rate reduction of 73.3% was noted for the vacuum-packaged cells.

## Discussion

Powdered infant formula is not a sterile product, and infantile infections of *C. sakazakii* are epidemiologically related to the consumption of contaminated, reconstituted PIF (Tall et al., 2017). *C. sakazakii* has a competitive advantage in dry environments due to its high tolerance to desiccation (Breeuwer et al., 2010), and the persistence of *C. sakazakii* in PIF during a 2.5-year period was reported (Barron and Forsythe, 2007). Some decontamination methods have been found by researchers to control the *C. sakazakii* in PIF, including increases or improvements in the traditional PIF processing method and reductions in the level of *C. sakazakii* through the use of plant-derived compounds during or after PIF reconstitution (Ha and Kang, 2014; Pina-Pérez et al., 2015; Shi et al., 2016); however, the impact of packaging and storage processes, important links in the PIF industrial chain, on the survival of *C. sakazakii* has not been explored. In this study, desiccated *C. sakazakii*, the intrinsic contamination of PIF in actual production processes, was simulated to study the influence of vacuum and air packaging followed by storage at different temperatures on the survival and environmental stress resistance of *C. sakazakii*.

We selected three different temperatures at which to detect the variation of the bacteria population within 10 days of storage: (1) 4°C was chosen to simulate the food freezing temperature, (2) 10°C was chosen as the transportation temperature, and (3) 25°C was chosen as a typical indoor temperature. At each of these three temperatures, both the tested packaging methods (vacuum and air) can lead to a decline in the amount of *C. sakazakii* in PIF (Figure 1). However, the vacuum packaging method produced a negative effect on bacteria survival more quickly compared with air packaging. For facultative anaerobes like *C. sakazakii*, it is generally recognized that a reduction in the oxygen level causes metabolic modification and a corresponding reduction in growth rate. Thus, the oxygen concentration in air is likely optimal for growth, and a reduction in the oxygen concentration to below 21% would presumably cause slower growth (Couvert et al., 2019). In agreement with our results, Duffy et al. (2001) reported that the packaging atmosphere has a noticeable effect on the growth rate of *Listeria* spp. (*L. monocytogenes* or *L. innocua*); *Listeria* on minced beef did not grow in vacuum-packed samples stored at 0 or 10°C, whereas *Listeria* spp. grew in aerobically packed samples at 10°C. The effect of vacuum packaging and air packaging on the survival of bacteria may depend on the state of the bacteria. Williams and Golden (2001) reported that for heat-injured *L. monocytogenes*, vacuum packaging at 4°C is not conducive to the survival of bacteria as compared with air packaging, but for uninjured cells, vacuum packaging at 4°C is more conducive to *L. monocytogenes* survival as compared with air packaging. The storage temperature may also affect the survival of bacteria under vacuum and air packing. Williams and Golden (2001) additionally found that for heat-injured *L. monocytogenes*, the survival of these bacteria at 20°C in vacuum packing is higher than that in air packing.

Our results show that the survival of *C. sakazakii* decreased at all three temperatures tested in this study (4, 10, and 25°C), but the survival rate decreased the fastest at 10°C. Lin and Beuchat (2007) monitored the survival characteristics of *E. sakazakii* in infant rice cereal for a period of 12 months and found that the population of *E. sakazakii* in cereal stored at 21°C decreased more quickly than that in cereal stored at 4°C. We saw a similar trend in the present study, where the amount of bacteria at 4 and 25°C decreased by 0.99 and 1.34 log CFU/ml, respectively, within 10 days. Also in agreement with our results, Al-Nabulsi et al. (2009) found that 21°C was more optimal for the survival of desiccated *C. sakazakii* compared with 10°C; the number of desiccated cells dropped from 4.35 to 4.15 log CFU/ml after 4 h at 21°C, whereas it decreased from 4.35 to 2.46 log CFU/ml at 10°C.

Trehalose, a non-reducing disaccharide of glucose, is assumed to play a pivotal role in the protection of *Cronobacter* spp. In dried stationary cells, the trehalose concentration was increased more than fivefold (Breeuwer et al., 2010). The accumulation of trehalose can protect proteins and cellular membranes from inactivation caused by a variety of stress conditions, including desiccation and cold (Elbein et al., 2003). Kandror et al. (2002) demonstrated that enzymes for trehalose synthesis are induced in *Escherichia coli* under cold-shock conditions and that the resulting accumulation of trehalose increases the cell viability when the temperature falls to near freezing. Therefore, we speculate that the accumulation of trehalose in desiccated *C. sakazakii* may have made the bacteria more likely to survive at 4°C than at 10°C.

*C. sakazakii* has a strong capacity to adapt to elevated osmotic pressure, low pH, heat, oxidation, desiccation, and bile salt (Alvarezordóñez et al., 2014; Fakruddin et al., 2014). These characteristics enhance the overall survival of *C. sakazakii* and increase the risk of contamination with these bacteria in dairy products. In the present study, the heat, acid, and bile salt resistance of *C. sakazakii* were determined to comprehensively evaluate the effect of different packaging methods on reducing PIF contamination with *C. sakazakii*. Some recent studies have assessed the effects of processing or pre-processing methods on the stress tolerance of bacteria. Exposure to 405 nm LED illumination was found to significantly enhance the susceptibility of *L. monocytogenes* and *Salmonella* spp. to simulated gastric acid (Li et al., 2018). *Trans*-cinnamaldehyde, the principal component present in cinnamon oil, reduced the tolerance of *C. sakazakii* to environmental stresses, such as heat, desiccation, acid, and osmolarity (Amalaradjou and Venkitanarayanan, 2011). Our results show that the percentage of survival for desiccated bacteria (without 10 days of storage) and for stored air-packaged or vacuum-packaged cells decreased during exposure to heat, acid, or bile salts. The vacuum packaging had the greatest impact on reducing the stress resistance of *C. sakazakii*, whereas desiccation without 10-day storage had the least impact. Our previous study demonstrated that desiccation stress significantly decreased the heat resistance of *C. sakazakii* in reconstituted infant formula (Shi et al., 2017). On the contrary, Chen and Jiang (2017) found that desiccated *Salmonella* cells in poultry litter showed enhanced heat resistance as compared to non-desiccated cells. And *rpoS* gene was involved in the cross-protection of desiccated *Salmonella* against high temperatures. Yang et al. (2015) reported that desiccation decreased the heat and acid resistance of *C. sakazakii* compared with unstressed cells. They speculated that the desiccation condition could induce the microorganisms to be metabolically exhausted, making it difficult for the cells to tolerate adverse conditions (Beales, 2004). The decreased environmental stress resistance of vacuum-packaged cells might be affected by the accompanying reduction in oxygen concentration, which could affect the normal metabolism of *C. sakazakii*.

## Conclusion

In conclusion, this study has analyzed the impact of storage temperature (4, 10, and 25°C) and packaging methods (vacuum packaging and air packaging) on the survival of *C. sakazakii* in PIF. While the impact of vacuum and air packaging at 10°C on the environmental stress resistance (heat, acid, and bile salt) of *C. sakazakii* has already been demonstrated, this is the first record of the impact of air and vacuum packaging on the survival and environmental stress resistance of desiccated *C. sakazakii*. Our results show that vacuum packaging significantly decreases the survival of *C. sakazakii* compared with air packaging, and the populations of air- and vacuum-packaged *C. sakazakii* stored at 10°C for 10 days decrease more than do similar populations stored at 4 or 25°C. Vacuum packaging significantly decreased the tolerance of *C. sakazakii* to heat, acid, and bile salt. Thus, our results suggest that the application of vacuum packing for PIF during shelf life could have a beneficial effect in minimizing the risk of *C. sakazakii* contamination in the reconstituted product. Vacuum packaging could be applied in the packaging step during manufacture of PIF or as a novel hurdle in food preservation in combination with other preservative technologies. This study provides a new perspective on choosing food packaging and processing methods by evaluating their impact on the environmental stress resistance of foodborne pathogens. However, further research is needed to evaluate the virulence properties of air-packaged and vacuum-packaged *C. sakazakii* and the ability of *C. sakazakii* to cause infection.

## Author Contributions

YB, HY, and CS conceived and designed the experiments. DG and SF performed the experiments. DG and YB analyzed the data. HY and DG contributed to reagents, materials, and analysis tools. YB and CS wrote the manuscript.

## Conflict of Interest Statement

The authors declare that the research was conducted in the absence of any commercial or financial relationships that could be construed as a potential conflict of interest.
